# Lysosomal Storage Disease-Associated Neuropathy: Targeting Stable Nucleic Acid Lipid Particle (SNALP)-Formulated siRNAs to the Brain as a Therapeutic Approach

**DOI:** 10.3390/ijms21165732

**Published:** 2020-08-10

**Authors:** Maria Francisca Coutinho, Juliana Inês Santos, Liliana S. Mendonça, Liliana Matos, Maria João Prata, Amália S. Jurado, Maria C. Pedroso de Lima, Sandra Alves

**Affiliations:** 1 Research and Development Unit, Department of Human Genetics, National Institute of Health Doutor Ricardo Jorge (INSA I.P), Rua Alexandre Herculano, 321, 4000-055 Porto, Portugal; juliana.santos@insa.min-saude.pt (J.I.S.); liliana.matos@insa.min-saude.pt (L.M.); sandra.alves@insa.min-saude.pt (S.A.); 2 Center for the Study of Animal Science, CECA-ICETA, University of Porto, Praça Gomes Teixeira, Apartado 55142, 4051-401 Porto, Portugal; 3 Biology Department, Faculty of Sciences, University of Porto, Rua do Campo Alegre, 4169-007 Porto, Portugal; mprata@ipatimup.pt; 4 CNC—Center for Neuroscience and Cell Biology, University of Coimbra, 3004-504 Coimbra, Portugal; liliana.mendonca@cnc.uc.pt (L.S.M.); mdelima@ci.uc.pt (M.C.P.d.L.); 5 CIBB—Center for Innovative Biomedicine and Biotechnology, University of Coimbra, 3004-504 Coimbra, Portugal; 6 i3S—Institute of Research and Innovation in Health/IPATIMUP—Institute of Molecular Pathology and Immunology of the University of Porto, Rua Alfredo Allen, 208 4200-135 Porto, Portugal; 7 University of Coimbra, CNC—Center for Neuroscience and Cell Biology, Department of Life Sciences, Calçada Martim de Freitas, 3000-456 Coimbra, Portugal; asjurado@ci.uc.pt

**Keywords:** lysosomal storage diseases (LSDs), neuropathy, substrate reduction therapy (SRT), RNA interference (RNAi), siRNA nanodelivery systems, stable nucleic acid lipid particles (SNALPs)

## Abstract

More than two thirds of Lysosomal Storage Diseases (LSDs) present central nervous system involvement. Nevertheless, only one of the currently approved therapies has an impact on neuropathology. Therefore, alternative approaches are under development, either addressing the underlying enzymatic defect or its downstream consequences. Also under study is the possibility to block substrate accumulation upstream, by promoting a decrease of its synthesis. This concept is known as substrate reduction therapy and may be triggered by several molecules, such as small interfering RNAs (siRNAs). siRNAs promote RNA interference, a naturally occurring sequence-specific post-transcriptional gene-silencing mechanism, and may target virtually any gene of interest, inhibiting its expression. Still, naked siRNAs have limited cellular uptake, low biological stability, and unfavorable pharmacokinetics. Thus, their translation into clinics requires proper delivery methods. One promising platform is a special class of liposomes called stable nucleic acid lipid particles (SNALPs), which are characterized by high cargo encapsulation efficiency and may be engineered to promote targeted delivery to specific receptors. Here, we review the concept of SNALPs, presenting a series of examples on their efficacy as siRNA nanodelivery systems. By doing so, we hope to unveil the therapeutic potential of these nanosystems for targeted brain delivery of siRNAs in LSDs.

## 1. Introduction

When, in 1998, Fire and Mello published their first discovery of double-stranded RNAs, which were able to trigger gene-silencing in *Caenorhabditis elegans*, thus acting as gene function regulators [1], no one realized we were looking at one of the last great advances in cell biology. Shortly thereafter, however, similar observations came from plants [2] and mammals [3,4]. The grounds were thus seeded for the establishment of the concept of RNA interference (RNAi) as a post-transcriptional regulatory mechanism that triggers gene-silencing through the action of small RNA molecules, coined small interfering RNAs (siRNAs). Soon, it became clear that this pathway could be elicited not only by the action of endogenous siRNAs, but also by chemically synthesized (RNA) molecules, exogenously introduced into the cells, as soon as they matched a series of criteria [5] (reviewed in [6]). Since then, RNAi has rapidly become one of the most powerful and widely used tools for the study of gene function, providing researchers with a potent and specific way to promote gene-silencing. However, RNAi’s true potential as a gene-silencing tool for therapeutic purposes is still being unveiled. Promisingly, RNAi-induced gene-silencing holds potential to hijack the inhibitory effects of conventional pharmaceuticals, which are mainly achieved by blocking their targets’ function. Additionally, it may also target a plethora of disease-related molecules that were previously considered “non-druggable”, as for example proteins that do not have enzymatic function, or whose conformation makes them (or their active sites) hardly accessible to conventional drugs [7].

Over recent years, the therapeutic potential of RNAi has been extensively assessed and clinical trials are ongoing for several disorders. Here, we discuss its application in a substrate reduction therapy for a group of genetic disorders characterized by intralysosomal accumulation of undegraded materials, where brain pathology remains a challenge even though some therapeutic approaches are available mainly for systemic pathology. As it happens with many other disorders, translation of one such approach into the clinical setting largely depends on the development of appropriate systems for targeted delivery, which allow for proper biodistribution and promote more favorable pharmacokinetics. Therefore, the development of such systems is of utmost importance. Here, we will review some of the latest advances on different nanotechnological platforms for siRNA delivery, with special focus on the so-called stable nucleic acid lipid particles (SNALPs), one of the most widely used lipidic nanocarriers presently, which combines a high nucleic acid encapsulation efficiency with the capacity of being engineered in order to promote targeting to specific cell surface receptors. Special attention will also be given to the targeting approaches towards the most problematic organ to treat—the brain. By doing so, we hope to unveil the therapeutic potential of these siRNA nanodelivery systems for targeted brain delivery in a series of rare but life-threatening genetic pathologies, which have been our research focus over recent decades, the so-called Lysosomal Storage Diseases (LSDs).

## 2. Lysosomal Storage Disorders

LSDs are inherited metabolic disorders that affect the biological function of lysosomes. In general, LSDs result from mutations in genes that code for lysosomal enzymes, transport proteins, activator proteins, or other gene products that are necessary for proper lysosomal function. Those mutations lead to substrate accumulation within the cell, which ultimately cause lysosomal enlargement, cellular dysfunction and systemic clinical symptoms (reviewed in [8]). Currently, there are almost 70 known LSDs and, even though the individual disorders are considered rare, their collective incidence may be as high as 1 in every 5000–8000 live births [9,10] (reviewed in [8,11]). These disorders are known for more than a century now, but their underlying pathophysiological mechanisms are yet to be fully understood. Traditionally, LSDs are grouped according to the nature of the primary storage material (reviewed in [12]). This classification relies on the biochemical data collected for early disease diagnosis and, according to it, LSDs are divided into sphingolipidoses, mucopolysaccharidoses (MPSs), glycoproteinoses, neuronal ceroid lipofuscinoses (NCLs) and glycogen storage diseases, among others. Although it has several limitations, this categorization has reached our days and it is still the most informative classification for those disorders resulting from a block of a single enzymatic step in a particular catabolic pathway. Nevertheless, it failed to accommodate other sorts of LSDs, such as those involving defects in the post-translational processing of lysosomal enzymes (e.g., mucolipidosis types II and/or III [13,14]) or the ones resulting from lysosomal membrane and transport defects (e.g., cystinosis [15]). For these cases, only the elucidation of their molecular basis allowed for a proper understanding and subsequent classification. Thus, the traditional LSDs categorization ended up being refined over the decades, in order to reflect the accumulating molecular knowledge, and accordingly, several diseases have been re-allocated to more appropriate sub-groups. Currently, the accepted classification relies on the molecular defects in the lysosomal system, as well as on the accumulating substrates. One such categorization focuses attention on both function and pathogenetic mechanisms, thus contributing to the development of logical forms of therapy (reviewed in [16]).

In general, LSDs clinical presentation often involves bone, muscle, liver, kidney, and spleen. Adding to lysosomal disease complexity and broad systemic involvement of multiple tissues and organs is the extensive neurological impairment that affects nearly two thirds of the cases. Determining whether lysosomal storage per se is a fundamental cause of neuronal dysfunction remains a challenge, and an important question to be answered. Neurons from different lysosomal diseases have been shown to present significant storage pathology quite early and yet survived for decades before accumulation eventually became cytotoxic. Still, this does not necessarily mean that those neurons remain functionally normal. Both the metabolic compromise and the presence of axonal, dendritic, and synaptic abnormalities, most likely, affect not only neurons, but also the neural networks in which they are involved. As a result, those networks are not optimally functioning even from early time points in the storage process. Eventually, the presence of such malfunctioning neurons is expected to reach a tipping-point at a systemic level, leading to clinical disease even in the absence of obvious neurodegeneration. Such clinical deficits would then be solidified and, most likely, worsened with the eventual death of neurons involved in those neural circuits (reviewed in [12]).

In recent years, biochemical and cell biology studies of LSDs have revealed an ample spectrum of abnormalities in a variety of cellular functions. These include defects in signaling pathways, calcium homeostasis, lipid biosynthesis and degradation and intracellular trafficking [17], but certainly there is still much to be learned on this subject. Interestingly, the more we become elucidated on the pathogenic cascades underlying lysosomal disease, the more intricate becomes the nature of the so-called “greater-lysosomal system” and its crucial role in cell homeostasis [18,19] (reviewed in [12]).

Whatever the mechanistic/physiological cascade triggering pathology, the burden of disease is tremendous, in most cases. LSDs critically affect well-being, life quality, and survival at all ages. Furthermore, even though our knowledge of the molecular and biochemical bases of these disorders has been increasing over the years, for most patients there is still no specific or curative therapy available [20].

### The Unmet Need—Defeating Central Nervous System Pathology

Of the about 70 described LSDs, more than two thirds are associated with cognitive or motor deterioration, demonstrating central nervous system (CNS) involvement [11,21,22]. This neurodegeneration may start early in life or not manifest until later, depending on the individual LSD and on the nature and/or extent of the enzymatic deficit. Still, once triggered, the progression of neurodegenerative signs is unrelenting (reviewed in [22]). The typical developmental pattern seen in virtually every “neuronopathic” LSD is one of regression. After a period of apparently uneventful progress, development slows, and peers start to acquire skills at an increasingly faster rate. Eventually, development reaches a plateau and acquired skills are then lost following the common pattern where the most recently acquired skills are lost first and the losses then progress in such a way that eventually the child becomes totally dependent on his/her caregivers (reviewed in [23]). Typically, diagnosis occurs post-symptomatically, when patients begin to show developmental decline after a period of normal development (reviewed in [8]). Some of the most common CNS manifestations include delayed development, spasticity in movement, hypotonia, macular cherry red spots, and seizures [24,25] (reviewed in [8]).

In general, the symptomatology of the so-called “neuropathic” LSDs is similar and, in the most severe cases, peripheral disease may remain subclinical because CNS deterioration is more progressive and severe than that of peripheral organs (reviewed in [8]). One representative example of this curious pattern is the phenotype of the so-called MPSs. MPSs are LSDs in which the major accumulating substrate is glycosaminoglycans (GAGs; previously known as mucopolysaccharides). This accumulation results from defects in the GAG catabolism. In fact, each MPS disorder is caused by a deficiency in the activity of a single, specific lysosomal enzyme required for GAG degradation (reviewed in [26]). These disorders are biochemically characterized by an accumulation of partially degraded GAGs within the lysosomes, and their subsequent accumulation in urine, blood, and cerebrospinal fluid (CSF) [27,28,29,30] (reviewed in [31]). As every other LSD, the MPSs are chronic and progressive syndromes that produce in multiple organ systems a spectrum of signs and symptoms that can differ substantially across different MPSs. In overall presentation, MPS I, II, and VII have many similar clinical features, even though hydrops fetalis is not generally seen in MPS I or II [32,33,34] (reviewed in [31]). Among patients who present the first symptoms after the neonatal period, the ones who suffer from the severe forms of MPS I, II, and VII have both somatic and cognitive involvement [31]. Still, there is a subtype of MPSs, which is characterized by an extremely severe neurological phenotype, although exhibiting little or no somatic involvement: the MPS III or Sanfilippo syndrome (reviewed in [26,35]). The characteristic feature in MPS III is that of a child who presents with normal development until the age of 12 to 18 months and then fails to develop normal speech. Initially, this is usually thought to be due to middle ear disease and deafness, but even when these problems are mitigated, speech fails to improve. Furthermore, developmental problems tend to increase during that period. Children with this disorder have only mild somatic abnormalities, which may hinder the diagnosis of an MPS disease. Still, once the children develop a challenging behavior that is typical of MPS III, the diagnosis becomes easier for an experienced physician. Such behavior is characterized by severe insomnia and extreme hyperactivity, which makes disease management extremely difficult. As the disease progresses, patients may also develop autistic behavior (reviewed in [35]). Then, skills are lost, and the children become unsteady and fall frequently, tending to develop neurological dysphagia. By their mid-teenage years most affected patients are dependent on their caregivers for all needs, before death occurs towards the end of the second or early in the third decade of life (reviewed in [23,35]).

Another classical example of the large phenotypic spectrum that characterizes LSDs is that of sphingolipidoses. Sphingolipidoses are a group of LSDs characterized by a defective metabolism of sphingolipids, which results in a decrease of the ceramide pool in lysosomes. Some of the most common and well-known LSDs, such as Gaucher disease (GD) or Fabry disease (FD), are sphingolipidoses, but this group includes a large number of other disorders: Metachromatic Leukodystrophy (MLD), Krabbe disease, Niemann Pick disease, GM1 and GM2 gangliosidosis. In sphingolipidoses, lysosomal lipid storage occurs both in the CNS and visceral tissues but CNS pathology is a common hallmark for all of them [11,36]. For GD, a prototype LSD, for example, even though there is a vast heterogeneity of disease manifestations among affected individuals, three major clinical entities/sub-types may be recognized (GD types 1, 2 and 3), with two of them being neuronopathic forms: type 2 GD and type 3 GD [37]. Briefly, type 2 GD is a lethal neuronopathic form, characterized by hypertonic posturing, strabismus, trismus, and retroflexion of the head shortly after birth. Visceral manifestations are also common, with massive hepatosplenomegaly and lung involvement. Type 3 GD is both a more heterogeneous and a more attenuated neuronopathic form, presenting in childhood with the pathognomonic sign of supranuclear horizontal gaze palsy [38].

Many other LSDs and sub-groups of LSDs may be listed that follow this general clinical pattern, where the most severe forms of the disease are the ones presenting CNS involvement, while the milder forms appear to be mostly somatic. Still, in recent years, pathophysiological boundaries have blurred between LSDs and other disorders including late-onset neurodegenerative diseases such as Alzheimer’s and Parkinson’s [12]. In fact, it is now well-established that heterozygous and homozygous mutations in *GBA1*, the gene involved in GD, are the highest genetic risk factor for Parkinson’s disease (PD). Moreover, a significant percent of PD patients have a mutation in one or more genes that cause a LSD, as recently shown by a whole exome study in PD patients [39,40]. This suggests that lysosomal loss of function is likely involved in PD pathology. It also sheds a different light over the general idea that there is an absence of neurological symptoms in the mildest forms of LSDs.

Finally, we cannot review the LSDs-associated neurological burden without referring to a particular group of LSDs known as NCLs or Batten disease, which are collectively considered to be the most common inherited neurodegenerative disorder of childhood. These are probably the neurological LSDs for excellence, but they may well also be those which remain most elusive. There are currently 13 genes known to cause NCLs. Most have an autosomal recessive inheritance pattern, but autosomal dominant inheritance can be seen in one of the adult-onset forms, CLN4. The mechanism of pathogenesis has been characterized for some disorders. For example, deficiency of the lysosomal enzyme palmitoyl-protein thioesterase 1 in the classic infantile form results in the inability to remove long-chain fatty acids from proteins, which impairs their normal breakdown. For other disorders, the pathogenesis remains unclear [41]. Indeed, even though the molecular basis of NCLs are now established, very little is known about the normal function of the disease-causing gene products. Therefore, it has also been quite a challenge to draw conclusions about how these are compromised by the disease-causing mutations identified so far. The characteristic pathology common to all these disorders is the accumulation of autofluorescence storage material within the lysosome and the widespread death of neurons. At a clinical level NCLs typically involve visual failure, medically refractory epilepsy and relentless declines in motor and cognitive skills, invariably ending in premature death. Each NCL is caused by mutations in a different gene, the so-called CLN genes. The first presentation of disease, which has been reviewed in detail elsewhere [42], varies between the different forms of NCL. Briefly, this tends to be a slowing in development followed by developmental regression in CLN1 disease, seizures in CLN2 disease, and loss of vision in CNL3 disease, just to sum up the most relevant ones. Typically, for individuals presenting early in life, disease progression is faster than that seen in those presenting later. However, there may be periods of plateau preceding subsequent deterioration [43].

Currently, effective treatments have been developed for a small number of LSDs, but the CNS pathology remains practically beyond reach. Up to now, almost all therapeutic approaches have been hindered by an inability to cross the blood-brain barrier (BBB) without invasive administration (intrathecal (IT) or intracerebroventricular (ICV)). Furthermore, even when (or if) the therapeutic molecules enter the brain, it is difficult to ensure therapeutic levels of enzyme distributed evenly throughout the entire parenchyma (reviewed in [8]). This is particularly relevant for enzyme replacement therapy (ERT), which remains the gold standard for LSDs treatment. In reality, no ERT is yet available that can cross the BBB to treat the primary CNS burden in the majority of LSDs (reviewed in [8]). CLN2 was the first and only LSD with an approved therapy directly targeted to the brain; all the other therapies address systemic non-neurological manifestations [44]. Still, even the successful CLN2 brain-targeted ERT is ICV administered, thus circumventing the inability of systemically administered recombinant enzymes to cross the BBB. Considering the previously referred MPS diseases, for example, there is now several available recombinant enzymes for MPS I, II, IVA, VI, and VII somatic manifestations. Also, for the sphingolipidoses subgroup of LSDs, several recombinant enzymes have reached the market with ERTs approved for GD and FD. Still, even when recombinant enzymes are available, there are some adverse events that may be associated with ERT, including infusion-related hypersensitivity reactions that can be characterized by flushing, headache, pyrexia, or urticaria. Furthermore, there is also a significant number of patients who develop IgE anti-drug antibodies [45,46,47,48,49,50]. In some cases, this leads to life-threatening anaphylactic reactions, which justify the need for ERT to be administered in a facility with appropriate medical support [51]. Therefore, there is still much to be done in order to improve the existing treatments and develop new and more effective therapies, which are able to cope with the neurological burden of these diseases (reviewed in [20]).

For ERT, for example, there are several possibilities being exploited from enzyme modification to ICV injection of the developed formulae. With regard to chemical modification of the lysosomal enzymes, deglycosylation has been the most widely tested as a method of increasing the serum half-life and, consequently, the therapeutic effect of lysosomal enzymes [52] (reviewed in [8]). Another chemical modification, which may be attempted to improve ERT efficacy is the covalent attachment of polyethylene glycol (PEG). PEGylation has also been employed to modify some undesirable properties for enzyme delivery, while allowing for the protein to maintain its activity [53] (reviewed in [8]). In fact, the simple addition of PEG to the therapeutic enzyme may be sufficient to significantly enhance its half-life, while increasing its size and aqueous solubility [54] (reviewed in [8]). There are some first (linear PEG) and second (branched PEG) generation PEGylated molecules already approved for clinical use, but not in the LSDs field. Adagen^®^ and Oncaspar^®^, are two first-generation PEGylated molecules used in leukemia treatment, while PEGIntron^®^, a second generation PEGylated drug is currently administered to treat hepatitis C (reviewed in [8]). Still, to the best of our knowledge, this technique has not yet been reported for lysosomal enzymes. The most promising method to expand ERT to neuronopathic LSDs by chemical modification involves the generation of a chimera through the fusion of the therapeutic enzyme with targeting peptides. Fusion of the recombinant enzyme to peptides, which are specifically recognized by cell surface receptors at the BBB endothelium, may actively increase cellular uptake and promote targeted brain delivery. Thus, over the last decade, there has been several teams creating either fusion proteins or conjugates with lysosomal enzymes for brain-driven ERT, although the in vivo success has been quite variable. For MPS diseases, for example, this approach has been extensively evaluated. Recently, Pardridge and co-workers have generated a fusion protein for the treatment of MPS IIIA, by adding a peptide targeting the human insulin receptor to a recombinant sulfamidase (the enzyme deficient in that pathology), which was intravenously (IV) injected into one male Rhesus monkey at a dose of 19 μg/kg. Importantly, 140 min post-injection, high amounts of the fusion protein were found in the total brain homogenate [55] (reviewed in [8]). Interesting results were also reported for MPS I, by another team developing different chimeras for the human alpha-L-iduronidase (IDUA), the enzyme deficient in that disorder [56].

Direct targeting of the CNS through ICV injections of recombinant enzymes is also being exploited. This method of treatment takes advantage of protein transport to the brain through the CSF via strategically placed catheters into the lateral ventricle. Although ICV injection is an established drug administration route for chemotherapy and antibiotics, in use for more than 50 years, it is typically a defined course of treatment administered by slow push bolus. Conversely, ERT is a lifelong treatment, which is anticipated to require ICV infusions administered on a dosing frequency based on the recombinant enzyme’s half-life. The chronic nature of one such treatment regimen poses unique challenges in achieving successful drug delivery and safe administration [57]. Still, the need for a treatment, which is indeed effective for the CNS manifestations of LSDs, has prompted several teams to evaluate its potential/feasibility. Thus, this sort of approach was attempted in vivo for several LSDs, including MLD, Sandhoff, CLN2 and MPS IIIB [58,59,60,61], suggesting its feasibility, although still being associated with a high risk of infection. For CLN2 in particular, the recombinant form of the enzyme whose activity is impaired in the disorder (recombinant human tripeptidyl peptidase 1, rhTPP1, commercially known as cerliponase alfa-Brineura^®^) has shown efficacy and tolerability in mouse and canine models of the disease when ICV delivered. Later, assessments of the CNS distribution of the enzyme in healthy cynomolgus monkeys demonstrated that rhTPP1 was able to reach deep brain structures such as the thalamus, midbrain, and striatum. Overall, the results from those studies in healthy animals and CLN2 disease models have helped the design of clinical trials in terms of administration, infusion times, and frequencies, as well as dosages. Soon cerliponase alfa reached clinical trials and its ICV injection lead to significant reductions in the rate of decline of motor and language functions in comparison with a natural history population. Based on the aforementioned clinical trial results, cerliponase alfa is now available in Europe for patients of all ages and in the USA for patients aged 3 years or over. Importantly, the enzyme is infused after antihistamine administration to reduce hypersensitivity reactions. In general, the procedure has been accepted by patients and families, and as yet, no patients have withdrawn from treatment for efficacy or safety reasons [62]. Still, it should be noticed that there are several risks which are known to be associated with ICV administration including infection, intracerebral hemorrhage, device failure, catheter malpositioning or obstruction, and CSF leakage [57]. Furthermore, both systemic and ICV enzyme delivery require weekly to monthly infusions as a lifelong treatment. Therefore, there is a real need for new and effective therapies to bypass the neurological dysfunction associated with LSDs.

Technology is evolving, though. Although ERT had initially set the trend, and even though many teams are still focused on making the best out of it, several other approaches have started being exploited for LSDs treatment. Some of them were granted orphan drug designations and a few have actually reached the market. Currently, four small molecules have already been approved for the treatment of different LSDs. Their mechanisms of action target the facilitation of subcellular transport [e.g., cysteamine (Cystagon^®^) for cystinosis] and the reduction of storage (miglustat (Zavesca^®^) and eliglustat (Cerdelga^®^), both approved for the treatment of GD). In 2018, the first pharmacological chaperone for an LSD has also reached the market: migalastat (Galafold^®^), which stabilizes misfolded forms of the enzyme alpha-galactosidase A, got FDA approval for the treatment of adults with confirmed FD and an amenable *GLA* variant [44].

The plethora of innovative ideas on pharmacological approaches is growing at an incredible rate and different mechanisms of action are being exploited from mutation-specific approaches such as stop-codon read-through [63,64,65] and splicing modulation with antisense oligonucleotides [66,67,68] to more generic approaches, such as the use of anti-inflammatory or neuroprotective drugs, which address endpoints of the LSDs pathophysiological cascade [69]. Not all these approaches hold potential to address brain pathology, but the neurological dysfunction has not been forgotten. Several strategies are being developed and evaluated. Ex vivo and in vivo gene therapy approaches and a variety of nanoparticle-based systems are currently under investigation in different labs having in view different diseases, and numerous studies have been conducted to optimize the different parameters required for proper treatment of LSDs with CNS involvement. A broad overview of all these investigations and the results of their application fall out of the scope of this review, but they have been extensively dissected elsewhere (see [8]). Also under consideration is a molecular approach based on RNAi to selectively down-regulate genes involved in the biosynthesis of accumulating substrates. This approach has been referred to as genetic substrate reduction therapy (gSRT) and is accumulating evidence on its feasibility in vitro over recent years [70] (reviewed in [71]). In the next section, we will further elaborate on this therapeutic approach and its supporting rationale, which will be grounded in the review of studies highlighting its potential towards clinical application.

## 3. RNA Interference (RNAi)—An Overview of the Silencing Machinery

**Figure 1:**
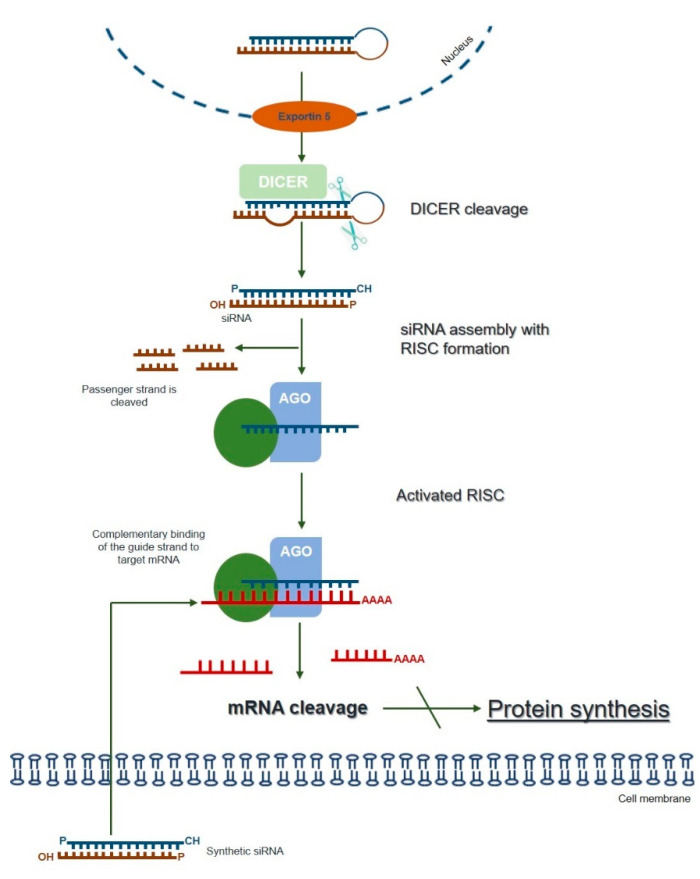
The mechanism of RNA interference (adapted from [7,73]). Long dsRNAs are produced within the nucleus and exported to the cytoplasm through exportin 5. Once they reach the cytoplasm, dsRNAs are cleaved by an enzyme called Dicer. The resulting fragments, consisting of a guide (antisense) strand and a passenger (sense) strand, are called small interfering RNAs (siRNAs). The siRNAs are then incorporated into the RNA-induced silencing complex (RISC), resulting in the cleavage of the sense strand of RNA by Argonaute 2 (AGO2), whereas the antisense strand guides RISC towards the complementary mRNA, which is cleaved into two mRNA fragments leading to the silencing of the target gene. The activated antisense–RISC complex can then be recycled for degradation of additional target mRNA molecules (see text for details). The RNAi-mediated gene-silencing can be triggered by synthetically produced siRNAs directly introduced into the cell, circumventing the Dicer-cleavage step. This shortcut reduces the potential for an innate immune interferon response.

The term RNAi was coined to describe a naturally and evolutionarily conserved mechanism of specific post-transcriptional gene-silencing, which can be mediated by different small RNA effector molecules, including endogenous microRNA (miRNA) and exogenous siRNA or short hairpin RNA (shRNA) [72] (reviewed in [7]). The endogenous siRNA pathway starts with the cytoplasmic cleavage of long double-stranded RNA (dsRNA) by an enzyme called Dicer. As a result, the fragments known as siRNAs are produced, which are then incorporated into the multiprotein RNA-induced silencing complex (RISC). At RISC, the sense (passenger) strand of siRNA is cleaved by the catalytic subunit of Argonaute 2 (AGO2), whereas the antisense strand serves to guide the RISC towards the complementary target mRNA sequence that is cleaved by AGO2 generating two mRNA fragments. The activated antisense strand-RISC complex can be recycled and can then move on to destroy additional target mRNA molecules. Ultimately, this process results in reduced gene expression at the levels of the target mRNA and the encoded protein (Figure 1; reviewed in [6,7,73]).

**Figure 2:**
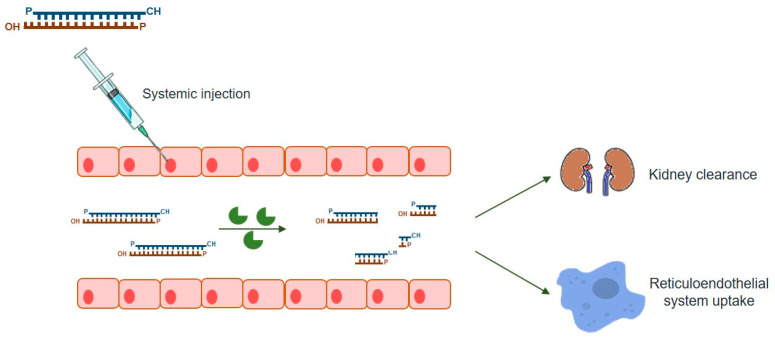
Barriers to the systemic delivery of siRNAs nanoparticles. In vivo, siRNAs systemic delivery faces several barriers, namely: stability in the blood stream; transport across the vascular endothelial barrier; diffusion through the extracellular matrix and endosomal escape. Upon systemic administration, nano-sized carriers might be rapidly cleared from bloodstream by the reticuloendothelial system (RES) and phagocytosed by the mononuclear phagocyte system (e.g., macrophages and liver Kupffer cells). These clearance processes, mediated by the interaction of the nanoparticles with blood components (e.g., immunoglobulins of the complement system), result in higher particle accumulations in RES organs, such as liver and spleen, relative to non-RES organs; therefore, decreasing the amount of nanoparticles that reaches organs such as the brain.

As mentioned above, this powerful gene-silencing process can be induced by exogenous siRNA through direct supply of synthetic siRNAs. Synthetic siRNAs have a well-defined structure mimicking that observed in vivo, which consists of a short (usually 21-bp) dsRNA with phosphorylated 5’ ends and hydroxylated 3’ ends with two overhanging nucleotides [3]. Alternative synthetic molecules do exist, including both shRNAs and miRNAs, which may also be chemically produced and elicit RNAi (reviewed in [7]). Compared to siRNA, an shRNA expression vector is more cost-effective than the bulk manufacturing of siRNA [74] while providing a more durable gene-silencing, as it is constantly synthesized in host cells [7]. On the other hand, endogenous miRNAs require only to be partially complementary to their target mRNAs 3’ untranslated region (3’-UTR), regulating the expression of their targets via all four ago proteins (AGO1-4), unlike the other molecules, which rely on AGO2 alone [75] (reviewed in [7]). Thus, one single miRNA may affect hundreds of different genes [74], thus exhibiting much more off-target effects than the other two types of molecules. siRNAs are, by far, the most popular RNAi-inducer molecules, with the great majority of studies relying on this sort of dsRNAs to promote targeted gene-silencing. Over the years, a series of empirical and rational guidelines started accumulating from the analysis of hundreds of functional siRNAs, paving the way to the current number of consensual rules one should follow in order to design an effective siRNA molecule [5]. There are also many websites and companies that either offer reliable methods for the design of effective siRNAs or design them on demand, which is understandable because given the small size of siRNAs, their chemical synthesis is not only relatively easy, but also rapid and scalable, so that several companies deliver them in ready-to-transfect format. This is, therefore, a simple, easy-to-handle RNAi-effector for virtually every lab need (reviewed in [6]). Nevertheless, as any other existing strategy, the use of siRNAs has also a series of drawbacks among which are the poor cellular uptake and instability under biological conditions that result in low bioavailability of naked siRNAs. In fact, unmodified siRNAs are quite stable under a variety of conditions in vitro but the same is not observed in vivo (Figure 2). When systemic delivery strategies are used, siRNAs are exposed to nuclease degradation being rapidly cleared from circulation, with consequent accumulation in kidney and fast excretion into urine [76,77,78]. In addition to circulating nuclease degradation and renal clearance, a major barrier to successful in vivo delivery of siRNAs is the uptake by the reticuloendothelial system (RES), whose physiological function is to clear foreign pathogens and remove cellular debris and apoptotic cells [79] (reviewed in [80]). Despite the phagocytosis role as a significant immunological barrier, not only in the bloodstream, but also in the extracellular matrix of tissues, unfortunately it does not act exclusively against viruses, bacteria, and fungi, being now well known that phagocytes are also highly efficient at removing several therapeutic macromolecules, including siRNAs [73].

Furthermore, studies developed by different teams have also demonstrated that siRNA molecules are not as safe as initially predicted (reviewed in [80]). Humans have evolved several host-defense mechanisms against dsRNAs, as they are feature of certain viral infections, which may hinder the use of synthetic naked siRNAs in vivo (reviewed in [73]). Evidence exists that high levels of siRNAs may trigger innate immune response via interaction with RNA-binding proteins such as Toll-like receptors (TLRs) and protein kinase receptor (PKR). Importantly, this process was shown to be both sequence- and length-dependent [81] (reviewed in [7,80]), implying that it can be, if not avoided, significantly reduced if the siRNA design takes this issue into account. It should be stressed that despite the promise of RNAi as a gene-specific process, the truth is that it can sometimes induce undesirable non-specific off-target effects, which raised the need to mitigate it through siRNA redundancy and/or chemical modification [21,82,83] (reviewed in [84]).

Still, the major hindrance in the development of RNAi therapies, as it happens with other approaches relying on nucleic acids, is the specific and efficient delivery of these molecules to their target cells. Since they are negatively charged and relatively large molecules, siRNAs do not readily cross the cell membrane, thus requiring endocytosis as the major route of entry into the cell, unless viral delivery is attempted (reviewed in [7]). The intracellular trafficking of siRNAs via the endocytic route usually begins in early endosomes. Then, those endosomes fuse with sorting endosomes, which in turn transfer their contents into acidified late endosomes (pH ~ 5.0–6.2). Subsequently, the late endosomes fuse with lysosomes that are further acidified (pH ~ 4.5), which contain several nucleases that may easily promote siRNA degradation. Thus, siRNAs that fail to be released from these acidic vesicles will ultimately be degraded [85] (reviewed in [7]). In addition, siRNAs need to escape to the cytosol, where RISC operates, so that they can exert their action, meaning that the endosomal membrane constitutes an important barrier that must be overcome. Owing to this, siRNA delivery strategies that take advantage of endocytosis must provide for endosomal escape (reviewed in [6,7]).

The successful therapeutic application of siRNAs requires the development of clinically suitable, safe, and effective delivery systems, and, therefore, the abovementioned issues should be considered to be key elements towards their rational design. As important as the effective silencing action, is the assurance that siRNAs are specifically delivered to their target cells at the desired concentration (reviewed in [86]).

## 4. RNAi as a Potential Therapeutic Approach for LSDs

Proof-of-principle on the potential of RNA-degrading technologies as effective tools to achieve significant reduction of the levels of accumulated substrate(s) in LSDs cell lines was first achieved by Diaz-Font et al., after assessing the potential of an artificially induced RNAi mechanism to reduce storage in one of the most common and well-known LSD: GD [87]. GD is a pan-ethnic LSD due to a deficiency in the lysosomal enzyme beta-glucocerebrosidase (GCase), which results in its inability to degrade glucosylceramide adequately. In the early 80s, purification of GCase and the subsequent cloning of the gene that codes for this enzyme (*GBA*) led to the pioneer works of enzyme replacement setting the grounds for ERT [88]. Later, SRT was also evaluated as a management therapy for GD, showing potential to also impact the neurological manifestations of the disorder [89]. Currently, there are two different small molecules approved for GD substrate reduction: miglustat, Zavesca^®^ (an amino sugar; reviewed in [90]) and eliglustat tartrate, Cerdelga^®^ (a longer-chain ceramide mimetic; reviewed in [91]). Still, in 2006, Diaz-Font and co-workers have also evaluated the potential of a siRNA-mediated strategy to promote substrate reduction in GD, thus establishing the concept of gSRT. In that work, the authors designed different siRNAs targeting the human *GCS* gene, transfected them into HeLa cells, having observed a clear reduction of *GCS* mRNA levels. They also confirmed inhibition at the protein level by assessing the enzyme GCS activity. Altogether, the results seemed promising and completely in line with the hypothesis that RNAi would be an effective method for genetic inhibition of GCS [87] (reviewed in [71]). By acting at the post-transcriptional stage of gene expression, siRNAs have a high degree of safety, as they do not interact with DNA, thus avoiding the mutation and teratogenicity risks of other gene therapy approaches, such as those involving plasmid DNA delivery [80]. Furthermore, a siRNA-based approach for SRT could also hold potential to overcome some of the well-known side effects of currently approved SRT drugs.

Importantly, GD is one of the few LSDs with approved formulae for both ERT and SRT, and chaperone therapy is also being tested for GD type 1. Although there are LSDs for which no therapy is yet available, a significant number of these disorders have at least one approved therapy. However, most approved drugs have a series of drawbacks, as reported in the previous section.

The MPSs subgroup of LSDs is among those in debt of either specific therapies (for several diseases that lack treatment approaches), or more efficient ones (for those with approved formulae presenting the above-referred limitations). Fortunately, the major biochemical pathways that give rise to GAGs (the substrates accumulating in this subgroup of diseases) are well known and almost all the involved genes have been cloned. Therefore, it seemed reasonable to design a similar RNAi-based substrate reduction approach for MPSs by targeting for degradation one (or more) of the enzymes responsible for GAGs biosynthesis. The underlying rationale for this idea prompted several investigators to target genes of the biosynthetic pathway that produce the different GAGs that accumulate in MPS diseases. Once again, their objective was to decrease GAGs synthesis by silencing specific genes, so that they would actively reduce substrate accumulation to slow down the pathology. This is particularly relevant as previous attempts to apply SRT for MPS diseases, particularly MPS III, had been focused on the use of non-specific inhibitors of GAG synthesis [92,93].

The first study aimed at impairing GAGs synthesis in MPS cells by using siRNAs was published early in this decade, by Dziedzic and co-workers [94]. This team transfected MPS type IIIA fibroblasts with four siRNAs targeting genes from the initial steps of GAGs’ biosynthetic cascade (*XYLT1*, *XYLT2*, *GALTI*, and *GALTII*). By real-time PCR, the authors observed a significant decrease in the mRNA levels of all targeted genes, which was accompanied by a significant reduction of their target protein levels. To further investigate the effects of the induced gene-silencing, the authors measured the incorporation of ^35^S sulphate in siRNA-transfected cells, to assess de novo synthesized amount of GAGs, having observed a considerable impairment in GAGs synthesis [94]. Later, they examined whether treating patients’ fibroblasts with two of those siRNAs together would be more effective than using single siRNAs in terms of GAGs synthesis inhibition. It was found that inhibition of GAGs synthesis in cells treated with two siRNAs was generally more effective than using single siRNAs; nevertheless, the differences were not statistically significant. Thus, the authors concluded that the potential benefit from the use of two siRNAs over the use of a single siRNA would be limited in the light of the cost-benefit ratio and possible stronger side effects of the putative therapy [95] (reviewed in [71]).

By the same time, Kaidonis and co-workers used RNAi technology to inhibit the expression of the *EXTL2* and *EXTL3* genes involved in the synthesis of heparan sulphate (HS), the glycosaminoglycan that accumulates specifically in MPS III [96]. Through a reporter gene assay, these authors showed that shRNAs directed to *EXTL2* significantly reduced endogenous target gene expression, while decreasing HS synthesis and reducing lysosomal storage of this GAG in an MPS IIIA cell line. Similar results were reached for at least one of the shRNAs designed to target *EXTL3*. Of note that this team has also assessed the incorporation of the studied shRNAs into a stable lentiviral expression system, with promising results in a reporter gene assay [96] (reviewed in [71]).

Later, Chmielarz and colleagues compared the effects of different treatment methods on the regulation of GAGs storage in various cell lines established from MPS I, MPS IIIA, and MPS IIIB patients [97]. Taking advantage of the existence of an approved ERT for MPS I, these authors addressed the effect of (i) ERT alone (laronidase; Aldurazyme^®^), (ii) different “anti-GAG” siRNAs alone, and (iii) the combination of both treatments on the above-referred cell lines. As anticipated, ERT was more effective than the siRNA treatment alone in reducing GAGs storage in all studied cell lines. Surprisingly, however, when a combined treatment was employed (ERT + “anti-GAG” siRNAs), results varied depending on the analyzed cell line(s), among which the combinatory effect of these two therapeutic approaches could be more or less pronounced than that obtained with either method alone. This was a particularly relevant observation, as it demonstrated that the efficacy of a combination of ERT and the use of siRNA depend on specific features of each cell line, thus highlighting that a combined therapy might potentially result in various effects in different patients [97] (reviewed in [71]).

More recently, Canals and his team [98] have also assessed the potential of four other siRNAs to act as gSRT triggers in MPS. They elected as targets the two genes that Kaidonis and co-workers, five years earlier, were able to inhibit, *EXTL2* and *EXTL3*, which code for two key proteins involved in the chain elongation of HS. However, instead of addressing the effect of gene-silencing in MPS IIIA, as the previous team did, Canals et al. analyzed MPS IIIC, another neurological MPS, in which HS specifically accumulates. After transfecting the siRNAs into fibroblasts from two different MPS IIIC patients, and assessing the effects from day 3 to day 14 after transfection, the authors observed a target mRNA inhibition of around 90%. Additionally, they have examined the downstream effect of that reduction, by quantifying the cell incorporation levels of a ^35^S sodium sulphate medium. The results were also promising showing that fibroblasts exhibited 30% to 60% decrease in the incorporation of ^35^S sulphate, 3 days post-transfection. Moreover, patients’ fibroblasts showed a significant reduction in HS accumulation, after a 3-day treatment with a siRNA for *EXTL2*, as revealed by an immunocytochemistry assay [98] (reviewed in [71]). Curiously, when the exact same molecules were tested in induced pluripotent stem cells (iPSC)-derived neurons, the same pattern was not seen [99,100]. This recent observation highlights the need for proper cell models for drug testing. In fact, only after an extensive in vitro assessment of the drug’s effect, should it be tested in vivo.

Altogether, these findings highlight the feasibility of a gSRT approach for MPS diseases, clearly demonstrating that positive effects on the levels of GAGs accumulation may be achieved whatever the step of the biosynthetic pathway one might target. This is very meaningful as it opens prospects for the use of a single compound to treat a series of pathologies (reviewed in [71]). Ultimately, the same principle may be applied for virtually all LSDs even though there are currently no reports attempting gSRT in other LSDs sub-groups. However, there are many hurdles that still need to be overcome since the therapeutic potential of one such approach depends on a series of factors apart from the efficacy of the siRNA itself. For example, the greater the residual enzymatic activity a patient retains, the greater is the likelihood for the patient to benefit from this approach (reviewed in [101]).

## 5. RNAi-Based Therapy Challenges

Most siRNA-based drugs currently being tested in clinical trials are directly administered to pathology-bearing regions, to avoid the complexity of systemic delivery. Still, the introduction of systemic routes for siRNA delivery is mandatory for this technology to achieve its full therapeutic potential. That is why many different teams worldwide have been focusing attention on this issue pursuing the development of suitable delivery systems for siRNAs to reach their sites of action. There are several possibilities being evaluated, from chemical modifications of the siRNA structure alone to specific siRNA nanodelivery systems. Whatever the case, an effective delivery system must fulfil a series of criteria, including biocompatibility, biodegradability, and non-immunogenicity, in order to enable multi-administration treatment modalities, which are crucial for improved clinical outcomes (reviewed in [102,103]). An ideal siRNA delivery platform should i) provide protection from blood nucleases and prolonged blood circulation, while avoiding rapid renal and/or hepatic clearance, ii) exhibit high siRNA loading abilities and be internalized by endocytosis, and finally iii), rely in siRNA with an endosome escape ability to reach the cytosol and enter the RNAi machinery, thus activating the RNAi pathway (reviewed in [6,80,104]).

### 5.1. Some Examples of Small Interfering RNAs (siRNAs) Delivery Systems

Early strategies for solving the dual problems associated with the intracellular delivery of siRNAs and their rapid excretion involved incorporation of siRNAs into lipid nanoparticles (LNPs), which are smaller and more homogeneous analogues of lipoplexes used for laboratory transfection [105,106,107] (reviewed in [108]). Lipoplexes (and other nanoparticles used for siRNA delivery) accumulate in the liver and other filtering organs, which limits their effectiveness in penetrating other tissues [107,109] (reviewed in [108]). Moreover, it has been shown that the administration of siRNAs via LNP delivery vehicles results in pro-inflammatory effects, because lipid-based vehicles can become entrapped in endosomes [110], where the TLR will recognize various moieties in dsRNAs, modified siRNAs or even their degradation products [111], eliciting an undesirable innate inflammatory response. Therefore, in most circumstances, the siRNA administration requires pre-treatment regimens, including antihistamines, non-steroidal anti-inflammatories, and even relatively high doses of glucocorticoids [112,113,114] (reviewed in [115]).

Many efforts were made to develop a class of nanoparticles that could circumvent these limitations. A plethora of polymers such as polyethylenimine (PEI), cyclodextrin, chitosan, dendrimers, and metal cores was used to develop novel and more stable nanoparticles, while ensuring those entities remained biodegradable, biocompatible, and non-toxic (reviewed in [7]). PEI, for example, is a synthetic polymer with extensive branches and dense cationic charge, which facilitates its complexation with siRNAs and protects them from RNAse degradation. This polymer has a high content of protonable amino groups, allowing it to act as a proton sponge to induce the release of siRNAs to the cytoplasm [78] (reviewed in [7]). Given these properties, PEI nanoparticles were initially viewed as promising vectors for siRNA delivery. Nevertheless, some wariness limits the PEI use in vivo, such as the weak electrostatic cohesion between PEI and siRNAs [116] (reviewed in [7]). To overcome this issue, the low molecular weight PEI was combined with other synthetic polymeric constructs such as PEG, and encouragingly the development of PEG-grafted nanocarriers led to significant advances in terms of vector efficiency [117]. The hydrophilic nature of PEG provides an aqueous shield around the nanoparticle surface, decreasing the extent of opsonization and, consequently, the recognition by the RES, thus resulting in an increase of the nanoparticle blood residence time (reviewed in [7,103]). Still, as works with PEG-grafted nanoparticles grew, the limitations involving these particles also started to accumulate. Early reports in clinical setting demonstrated that upon subsequent administrations of PEG-grafted nanocarriers (PEGylated LNPs in particular), an immune response could be elicited, leading to a rapid blood clearance of the nanoparticles, which inexorably compromised their clinical utility [118,119] (reviewed in [103]). It became further known that PEGylation also exerts a negative effect on cellular internalization and endosomal escape, significantly hindering the cellular uptake of LNPs and the escape of their entrapped siRNAs, because it limits the contact between membranes [120,121] (reviewed in [103]). Recently, however, the use of internalizing ligands has also contributed to the emergence of novel lipid-based nanoparticles with remarkable transfection efficacy [103]. Among them is a subclass of lipid-based nanoparticles called SNALPs, one of the most widely used LNPs presently [80].

### 5.2. Stable Nucleic Acid Lipid Particles (SNALPs)

**Figure 3:**
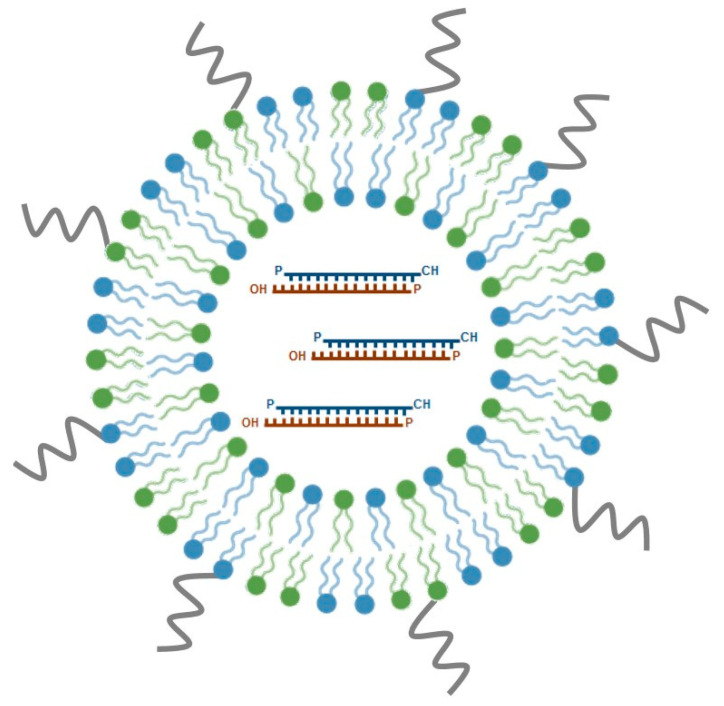
Schematic representation of stable nucleic acid lipid particles (SNALPs). SNALPs are a particular class of lipid nanoparticles (LNPs), which comprise cationic lipids (in blue), non-ionic lipids (in green), and polyethylene glycol (in grey; see text for details). SNALPs are nanoparticles, displaying optimal physico-chemical features for in vivo delivery of therapeutic nucleic acids, such as siRNAs. siRNAs are enclosed in the hydrophilic core of the nanoparticle. These siRNA-lipid particles show considerably enhanced cellular internalization and endosomal escape of siRNA. Several SNALP-formulated siRNA therapeutic molecules have reached clinical trials and are currently being evaluated for efficacy. The most well-known example is Patisiran^®^, the first FDA-approved siRNA drug.

SNALPs are among the most promising nucleic acid delivery nanosystems for in vivo application and have emerged as one of the leading lipid-based siRNA formulations under clinical development. Typically, SNALPs have a mean size of 100 nm and a neutral charge, comprising, three important components: an ionizable cationic lipid, a neutral helper lipid and a PEG-derivatized lipid (Figure 3). These PEG stabilized LNPs have been originally developed by Tekmira Pharmaceuticals Corporation, currently Arbutus Biopharma (http://www.arbutusbio.com/our-science/lnp-delivery-platform.php) and have been gaining momentum as more and more data on their in vivo efficacy accumulate. The underlying rationale for the development of these particles was to combine the potential of a fusogenic and ionizable cationic lipid, crucial for efficient encapsulation of siRNAs at acidic pH, with that of helper lipids (1,2-distearoyl-sn-glycero-3-phosphocholine (DSPC) and cholesterol), to stabilize the lipid bilayer, and add PEG-derivatized lipids, which would provide an aqueous shield around the nanoparticle surface, thus promoting prolonged blood circulation. Currently used cationic lipids include DODAP, DLinDMA and, more recently, DLin-KC2-DMA, whereas diacylglycerols (DAGs) are the most frequently used PEG-derivatized lipids (reviewed in [103]).

Over the last 15 years, many data on the efficacy of SNALP-formulated siRNAs for targeted gene-silencing in different models in vivo, were produced. The first study to demonstrate a specific silencing effect of SNALP-formulated siRNAs was performed by Morrissey and co-workers [122], and involved the targeting of the hepatitis B virus (HBV) in an in vivo mouse model of HBV replication. The SNALP-formulated anti-*HBV* siRNAs were administered by IV injection into mice carrying replicating HBV, according to a dose regimen of three daily IV injections of 3 mg/kg/day, and its efficacy was compared to naked siRNAs. A reduction of serum HBV levels by at least one order of magnitude was observed, and, most importantly, this reduction was specific, dose-dependent, and lasted for up to 7 days after administration. Furthermore, the application of SNALP-formulated siRNAs resulted in a clear increase of the half-life in plasma and liver, when compared with naked siRNAs. Although they are unmodified, naked siRNAs were rapidly eliminated from the plasma compartment (elimination/circulation half-life (T1/2) of ~ 2min), chemically modified siRNAs had a slightly increased half-life (T1/2 ~ 49 min). Whatever the case, the half-life values of naked siRNAs were dramatically different from those observed when the same siRNA was given as a SNALP-formulated drug (T1/2 ~ 6.5 h). Similarly, SNALP-formulated siRNAs exhibited lower toxicity and immunostimulatory side effects than their unformulated counterparts [123] (reviewed in [73,103]).

Shortly thereafter, Zimmermann and colleagues [124] came up with the first report on the efficacy of SNALP-formulated siRNAs systemic delivery in non-rodent species. This team encapsulated siRNAs against apolipoprotein B (ApoB) in SNALPs and administered that formulation by IV injection to cynomolgus monkeys at doses of 1 or 2.5 mg/kg. Apo B is essential for the assembly and secretion of very-low-density lipoprotein (VLDL) and low-density lipoprotein (LDL), which are required for the transport and metabolism of cholesterol. Elevated ApoB and/or LDL levels are long known to correlate with increased risk of coronary artery disease [125]. To address the therapeutic potential of SNALP-formulated anti-*ApoB* siRNAs, Zimmermann et al. evaluated the pharmacokinetics, efficacy, and safety of that formulation, finding out that a single SNALP-formulated siRNA injection resulted in dose-dependent silencing of ApoB mRNA expression in the liver (where it is predominantly expressed), with maximal silencing of more than 90%. Results were visible as early as 48 h post-administration and persisted for 11 days at the highest administered dose [124]. Interestingly, the silencing effect of SNALP-formulated siRNAs was over 100-fold more potent than that observed for cholesterol-conjugated anti-*ApoB* siRNAs, which the authors had already proven to be efficient for in vivo silencing of ApoB in rodents [124,126]. It was also shown that the degree and persistence of RNAi-mediated silencing in cynomolgus monkeys far exceeded the results obtained with rodents. The authors proposed that the longer duration observed in primates could either relate to species differences in the efficiency and stability of the RISC or to the mitotic state of the hepatocytes analyzed. Finally, treatment efficacy was confirmed by measuring the expected downstream effects of ApoB mRNA reduction: both plasma ApoB-100 protein and serum cholesterol levels decreased. Overall, results were consistent with an immediate, potent, and lasting biological effect of SNALP-formulated anti-ApoB siRNAs, supporting RNAi-based therapeutics as a potential new class of molecular pharmaceutical drugs and further validating SNALPs as efficient siRNA nanodelivery systems [124] (reviewed in [73,103]).

By the same time, the team of Geisbert focused efforts on assessing the potential of RNAi against a lethal Ebola virus (EBOV) challenge, with similar success. The EBOV infection causes a frequently fatal hemorrhagic fever (HF) that is currently refractory to the available antiviral therapeutics. Therefore, RNAi came up as a possible treatment option, as it held potential to specifically target the EBOV genome inhibiting its expression. Proof-of-principle on this was achieved when guinea pigs were treated with four different siRNAs targeting the polymerase (L) gene of the Zaire species of EBOV (ZEBOV), formulated in SNALPs, either before or after lethal ZEBOV challenge. Complete post-exposure protection against a lethal ZEBOV was revealed, both in guinea pigs [127] and Rhesus monkeys [128] injected with SNALP-formulated siRNAs, specifically designed to target different regions of the non-infectious ZEBOV RNA genome. In these two works, the animals were subjected to repeated administration of the SNALP-formulated siRNAs according to different dose regimens: for guinea pigs, the authors used 1 mg/kg per dose on days 1 to 6 after viral infection [127]; for macaques, the authors used 2 mg/kg per dose on days 1, 3, and 5 after challenge with ZEBOV [128]. Complete post-exposure protection against the virus clearly showed that the RNAi-based therapeutic approach employed could hold promise for the treatment of people infected not only with ZEBOV HF but probably also with other emerging viral infections [128] (reviewed in [73,103]).

Another outstanding study, on the efficacy of in vivo silencing promoted by SNALP-formulated siRNAs aimed at confirming the RNAi-mediated mechanism of action of siRNA-based cancer therapeutics in mice was performed by Judge and colleagues. To establish proof that systemically administered siRNAs could elicit RNAi-mediated anticancer efficacy in the absence of significant immune activation the authors selected two cell cycle protein kinases known to be cancer targets with well characterized mechanisms for direct tumor cell killing: kinesin spindle protein (KSP) and polo-like kinase 1 (PLK1) [129]. Previously, it had been shown that inhibition of both KSP and PLK1 rapidly resulted in mitotic arrest and apoptosis induction [130,131,132], making these two kinases ideal targets for RNAi silencing in tumor cells. In the work of Judge and co-workers, mice bearing established Hep3B orthotopic liver tumors were subjected to IV administration of 2 mg/kg of SNALP-formulated anti-*PLK1* siRNA twice a week for three weeks. An equivalent dose of SNALP-formulated anti-*KSP* siRNA was used in a hepatic Neuro2a tumor mouse model. Both experiments resulted in significant suppression of tumor growth, but not in its complete eradication. Still, it is important to note that this treatment schedule was devoid of immune responses. Since the well-designed methodological approach followed by the authors allowed to discriminate siRNA-mediated specific effects on gene expression from off-target effects, Judge et al. succeed to demonstrate that systemic administration of SNALP-formulated siRNAs can actually trigger RNA-mediated cleavage of target mRNAs within solid tumors, silencing their expression at a magnitude sufficient to induce mitotic disruption and subsequent apoptosis of tumor cells [129] (reviewed [103]).

The success obtained in these different works prompted additional studies on the use of RNAi as a therapeutic tool, which soon will boost the clinical evaluation of SNALP-formulated siRNAs. From the above-referred formulations, a significant number has moved into clinical trials and its therapeutic potential has been further evaluated, namely for those addressing hypercholesterolemia, Ebola virus infection, and liver cancer (reviewed in [73,103]).

Currently, patisiran (ONPATTRO^TM^) is the only commercially available siRNA drug formulated into SNALPs. Patisiran is an RNAi therapeutic agent that specifically inhibits hepatic synthesis of transthyretin (TTR) by targeting a genetically conserved sequence in the 3’ untranslated region of mutant and wild-type TTR mRNA. Clinical trials performed in both healthy adult volunteers and patients with hereditary TTR-mediated amyloidosis (hATTR), have shown that patisiran was able to consistently and specifically reduce serum TTR protein levels and tissue TTR protein deposits (known as amyloid), the hallmark of the pathology. Interestingly, the siRNA oligonucleotide molecule that ended up gaining approval for the treatment of hATTR in adults with stage 1 or 2 polyneuropathy was tested in two independent phase I trials, which involved drug encapsulation into two different lipid-based nanocarriers: ALN-TTR01 and ALN-TTR02 (patisiran). ALN-TTR01 was encapsulated in the first-generation version of LNP carriers, while ALN-TTR02 was encapsulated in a SNALP. The results from both trials were published in 2013 in the New England Journal of Medicine [112]. To summarize the published reports, each of the two formulations was studied in a single-dose, placebo-controlled phase I trial. Both formulations showed rapid, dose-dependent, and durable RNAi-mediated reduction in blood TTR levels. Yet, ALN-TTR02 was much more potent than ALN-TTR01, hence being the chosen formulae, which moved on into phases II and III [113,133,134] clinical trials. In all the reported studies, patisiran reduced serum TTR protein levels, regardless of age, race, sex or TTR mutation, and exhibited linear and time-independent pharmacokinetics with multiple-dosing. Most importantly, the drug did show therapeutic effect, significantly improving the pathological manifestations and quality of life of the patients (aged 24 to 83 years) with hATTR neuropathy that participated in the phase III study, as compared with placebo. The most frequent adverse events reported were peripheral edema and infusion-related reactions (reviewed in [135]). Taking together all these positive results, on August 2018, patisiran received its first global approval for the treatment of the polyneuropathy of hATTR in adults in the USA (https://www.drugs.com/history/onpattro.html) and, subsequently, in Europe, becoming the first, and so far the only, approved RNAi therapeutic agent.

Overall, the studies reviewed above aimed at developing efficient methods for siRNA systemic delivery. Yet, for gene-silencing-based drugs such as siRNAs to pave their way into the clinical practice, another important issue to deal with is how to endow the delivery system with targeting capacity.

### 5.3. Ligand-Mediated SNALP Targeting

**Figure 4:**
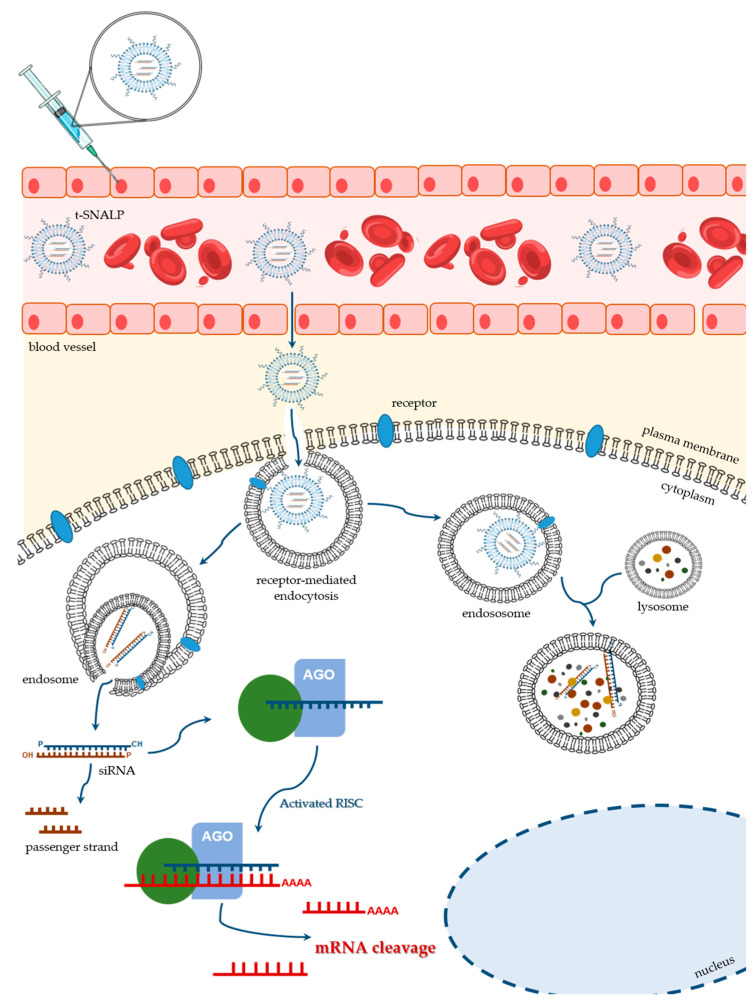
Schematic representation of the ligand-mediated SNALP targeting for delivery of therapeutic siRNAs. After being injected into the bloodstream, t-SNALPs must cross the endothelial barrier and diffuse through the extracellular matrix. When reaching their target cells, the engineered ligands, which are covalently attached to the SNALP surface, interact specifically with their receptors on the plasma membrane. That interaction will result in the t-SNALP internalization through receptor-mediated endocytosis, for example. Once inside the cell, siRNAs must escape the endocytic pathway (right side of the picture) and avoid lysosomal destruction. Instead, siRNAs need to be unpacked and released into the cytoplasm, where they can be incorporated into the RNAi machinery (left side of the picture) and actively promote the silencing of their target gene(s).

The nanotechnological platforms for siRNA delivery have been widely tested in the oncobiology field, with data on the efficacy of different drug delivery systems arriving at a tremendous velocity. RNAi technology has been the gold standard to achieve down-regulation of several overexpressed genes in cancer. In this context, improved targeting specificity and enhanced delivery efficacy have deserved a special attention. Some of the recent studies in this domain explore the application of SNALPs. Although SNALPs can exhibit passive accumulation into tumors, due to their large fenestrated endothelium, which results in an enhanced permeability and retention effect [136] (reviewed in [137,138]), big improvements can be achieved through the covalent attachment of internalizing targeting ligands that will specifically interact with their receptors usually overexpressed on the surface of target cells (Figure 4) [103]. It is understandable that several teams, including our own, have invested on the development of different tumor-targeted SNALPs, by coupling specific moieties to the surface of those PEGylated lipid nanocarriers.

In this regard, we have assessed the efficacy of transferrin receptor (TrfR)-targeted SNALPs encapsulating gene-silencing tools for chronic myeloid leukemia (CML) treatment [139]. CML is a hematopoietic stem cell disease, whose molecular trigger is the *BCR-ABL* oncogene. This gene encodes the Bcr-Abl protein, which is a constitutively active tyrosine kinase, responsible for the malignant transformation in the disease [140]. Thus, *BCR-ABL* seemed a good target for the application of gene-silencing approaches, using either siRNAs or antisense oligonucleotides (ASOs). The question was then how tumor cells could be targeted to selectively deliver the gene-silencing tools. We chose to couple transferrin (Trf) to th e surface of SNALPs encapsulating either anti-*BCR*-*ABL* siRNA or anti-*BCR-ABL* ASOs. Trf is an 80 kDa glycoprotein that undergoes internalization by endocytosis mediated by TrfR, a cell membrane associated glycoprotein (reviewed in [141,142]), which is known to be overexpressed in tumor cells [143,144]. After extensively characterizing the developed targeted Trf-SNALPs in terms of size, encapsulation yield, amount of coupled Trf protein and size stability over time, having registered very satisfactorily reproducible results, we moved on to address the selective internalization of the Trf-SNALPs, and clearly demonstrated that it occurred through TrfR-mediated endocytosis. Finally, the capacity of those formulations to achieve the desired therapeutic effect was also evaluated and a significant correlation between the reduction of *BCR-ABL* mRNA levels and the expected decrease of Bcr-Abl protein expression and cytotoxicity was observed [139]. Following these findings, we performed further studies aiming at generating Trf-SNALPs co-encapsulating anti-*BCR-ABL* siRNAs and imatinib. Imatinib is the first-line treatment for CML, which operates by effectively blocking Bcr-Abl activity (reviewed in [145,146]). Since many CML patients present with resistance and intolerance to imatinib, siRNAs and imatinib were co-encapsulated at different molar ratios. The developed Trf-SNALPs enabled the encapsulation of both siRNAs and imatinib at molar ratios that allowed reaching therapeutic doses, which clearly resulted in increased anti-tumoral activity [147]. This sort of approach is particularly attractive, since it allows triple targeting, while using a single therapeutic agent: the developed Trf-SNALPs are targeted to TrfR at the cancer cell surface, addressing two different molecular targets, BCR-ABL mRNA (through the use of siRNAs) and Bcr-Abl protein (through the use of imatinib) [147].

Similar efforts were conducted to design siRNA-containing SNALPs targeted to cancer cells and their tumor microenvironment. Actually, the aggressiveness of a tumor does not rely on the cancer cells alone, but rather on the cross-talk between cancer cells and other cells from the tumor microenvironment such as the endothelial cells (reviewed in [148]). To achieve such goal, we have covalently attached the F3 peptide to those nanoparticles with the rationale to target not only the cancer cells, but also angiogenesis. In fact, the F3 peptide is internalized upon specific binding to nucleolin, a receptor that is overexpressed on the surface of both cancer cells and tumor blood vessel cells [149,150]. Overall, our work culminated in the development of F3-SNALPs, whose general features were adequate for systemic delivery and led to a significant improvement in their internalization by both cancer and endothelial cells from angiogenic blood vessels, which was further correlated with effective gene-silencing [151,152]. Soon after, we have also assessed the therapeutic potential of those F3-SNALPs, upon encapsulation of a siRNA against *PLK1* [153] in prostate cancer (PC3) and angiogenic endothelial (HMEC-1) cells, having observed a significant decrease in cell viability, which was mediated by a marked *PLK1* silencing, both at the mRNA and protein levels. We have evaluated the anti-*PKL1* F3-SNALPs effect on the sensitization of cancer cells to conventional chemotherapy (paclitaxel), having observed the ability of anti-*PKL1* F3-SNALPs to render cancer cells sensitive to paclitaxel, thus potentially contributing to lower undesired side effects and resistance of cancer cells to conventional chemotherapy [153]. In another work, we have also designed a F3 peptide-targeted liposomal strategy, involving sterically stabilized pH-sensitive liposomes targeting the cell surface nucleolin, and exploited its potential for delivery of doxorubicin (DXR) to cancer cells and the tumor microenvironment (endothelial cells) in breast cancer, with similar success [154]. Later, the same liposomal strategy was successfully applied for the simultaneous delivery of a synergistic combination of the pro-apoptotic csc-ceramide (C6-Cer), an inhibitor of PI3K/Akt signaling, and DXR, aiming at promoting tumor cell death towards breast cancer treatment [155,156]. Finally, since an effective treatment for breast cancer would require targeting both cancer stem cells (CSCs) and non-stem cancer cells (n-SCCs), as the second may also originate CSCs through epithelial-to-mesenchymal transition [157], Fonseca et al. showed that F3-functionalized pH-sensitive liposomes were able to target simultaneously nucleolin-overexpressing CSCs and non-SCCs [156].

We have developed similar targeted approaches using peptides coupled to liposomal systems towards overexpressed tumoral receptors for treatment of other cancer types such as glioblastoma (GBM). For that purpose, chlorotoxin (CTX), a scorpion-derived peptide reported to bind selectively to glioma cells, while showing no affinity for non-neoplastic cells, was covalently coupled to SNALPs encapsulating either siRNAs or ASOs [158], the latter being designed to inhibit miR-21 which is overexpressed in the majority of tumors, including GBM. We have shown that targeted SNALP-mediated miR-21 silencing in cultured GBM cells promoted tumor suppressor expression and caspase activity, and reduced tumor cell proliferation [158]. Biodistribution analysis of IV-administered CTX-coupled SNALPs into GBM-bearing mice has revealed that these targeted nanoparticles preferentially accumulate within intracranial tumors, while sparing healthy tissue [158]. Moreover, systemic delivery of CTX-coupled SNALPs-formulated anti-*miR-21* oligonucleotides led to efficient release of the encapsulated nucleic acids into the brain tumors, which was associated with a significant and specific miR-21 silencing, resulting in increased mRNA and protein levels of its target, RhoB (Rho-related GTP-binding protein RhoB), while showing no signs of immunogenicity [159]. Importantly, CTX-coupled SNALP-mediated miR-21 knockdown combined with oral administration of the tyrosine kinase inhibitor sunitinib resulted in significant decrease in tumor size, cell proliferation and angiogenesis, enhanced apoptosis, and improvement of animal survival. No evidence of acute cardio- and hepatoxicity, potentially induced by the treatment as side-effect, was found, thus revealing that this multimodal approach holds great promise towards GBM therapy [159].

Overall, the reported studies highlight not only the potential of targeted lipid-based nanosystems, including SNALPs, to promote efficient and specific intracellular delivery for a whole new generation of molecular drugs, but also the importance of choosing the most adequate target receptor for each disorder and, consequently, the moiety to be coupled to the nanocarrier surface in each case. This sort of technology may also bring a hope for several diseases that urgently need innovative and effective therapies, such as the life-threatening LSDs.

## 6. General Considerations on the Pre-Clinical Development of RNAi-Based Substrate Reduction Approaches for LSDs

Currently, there is a lack of studies on the development of delivery strategies for siRNA-triggered SRT, even though this is a critical issue that must be carefully addressed whenever designing a therapeutic approach based on the use of siRNAs or other antisense technologies. This is not surprising, since delivery optimization is often left till the later stages of pre-clinical development (often following rigorous sequence optimization). However, it is fundamental to address this issue earlier as it is instrumental to a successful clinical translation [160]. This is especially meaningful for neurological diseases, as the BBB is one of the major challenges for targeted drug delivery. As it happens with many other disorders, translation of any siRNA-based approach for neurological LSDs into the clinical setting largely depends on the development of appropriate delivery systems, which allow for proper biodistribution and promote more favorable pharmacokinetics, while ensuring that the drug reaches specifically its therapeutic target.

Characterized by their favorable features for successful in vivo application, including prolonged blood-circulation times, SNALPs are highly bioavailable and may represent excellent nanodelivery systems for siRNA-based drugs, as referred in the previous section. Furthermore, SNALPs surface may be engineered with internalizing ligands targeting specific receptors, expressed in different cell types, including brain cells, which may allow for the simultaneous systemic and brain delivery. In fact, there are several types of receptors, expressed on the capillary endothelium of the brain, such as Trf, LDL, insulin, and nicotinic acetylcholine (nACh) receptors, and any moiety targeting one of these receptors may hold potential to promote receptor-mediated endocytosis (RME) of therapeutic compounds [161]. Still, caution is needed when choosing the moiety to be tested in each case. Trf, for example, is an ion transporting protein interacting with receptors ubiquitously expressed in various tissues (reviewed in [142]) and has been shown to promote cell uptake of lipid-based nanosystems in a multitude of cells, including neurons [162,163,164,165,166,167]. Moreover, we have proposed that Trf can trigger the cytoplasmic delivery of the carried nucleic acids by destabilizing the endosomal membrane, enhancing transfection [162,163,168]. Trf-associated cationic liposomes were initially assessed with great success, in our laboratory, for their potential to deliver plasmid DNA in a plethora of cells, including cultured cancer cells and primary neuronal cultures, as well as into the rat brain upon intracranial injection [166,167]. Later, their capacity for siRNA delivery was also assessed, with very promising results [168]. In fact, when we complexed Trf-associated cationic liposomes with siRNAs, at different lipid/siRNA (+/−) charge ratios, and assessed the cellular internalization of the resulting Trf-lipoplexes, confocal microscopy analysis showed fast uptake via endocytosis. Furthermore, in GFP-expressing glioblastoma cells, Trf-lipoplexes showed enhanced gene-silencing with minimum cytotoxicity in comparison to Trf-free lipoplexes [168]. Evidence was provided for the feasibility of Trf-lipoplexes to promote efficient down-regulation of the transcription factor c-Jun without detectable off-target effects, both in neuronal cell lines [168] and primary cultures of cortical neurons [169,170], which resulted in significant neuronal protection and repair from glutamate-induced damage or oxygen/glucose deprivation [168,170]. Importantly, upon stereotactic injection of anti-*c-Jun* siRNAs formulated into Trf-lipoplexes in a rat model of excitotoxic brain lesion, efficient and specific gene-silencing was achieved with minimum toxicity, leading to significant improvement in neuronal survival and attenuation of the inflammatory reaction, characterized by microglia activation and gliosis, caused by the induced excitotoxic damage [170]. Seemingly, whereas Trf would be an auspicious ligand to promote therapeutic delivery in LSDs, Trf-SNALPs would be an attractive choice of nanoparticles for targeted siRNA delivery in these multisystemic neurodegenerative disorders.

Other potential ligands for targeted delivery of “anti-LSDs” drugs are the rabies virus glycoprotein (RVG)-derived peptides, which hold promise to facilitate drug delivery into the CNS. Son et al. have even described RVG as a potential “magical bullet” for the targeting of genes to the brain [171]. Knowing that the RVG is a 505 amino acid glycoprotein responsible for the neurotrophic nature of the rabies virus infection, peptide derivatives of RVG, such as rabies virus derived peptide (RDP) and RVG-29, are emerging as promising targeting ligands for brain delivery (reviewed in [172]). The RDP is a 39 amino acid derivative of residues 300–357 of RVG that functions as an important nerve binding region [173]. It retains the neurotrophic penetrating properties of RVG, acting as a facilitating ligand for neural cell entry of a conjugated payload. Although there is still conflicting evidence in the literature regarding which receptor of the BBB is used by RVG and its derivatives, several studies point out an interaction between RVG and nicotinic acetylcholine receptor (nAChR). This interaction is thought to be the major responsible for BBB-crossing properties of RVG, but there is evidence on its association with other neural cell-specific molecules, such as neural cell adhesion molecule (NCAM) and Neurotrophin receptor p75 (p75NTR) (reviewed in [172,174]). Furthermore, interactions of RVG with carbohydrates, gangliosides, and lipids, also hold a stake in its entry into the CNS (reviewed in [174]). Whatever the case, the exact mechanism by which the RVG peptide traverses the BBB and enters cells is yet to be elucidated [175]. However, no doubts exist: nAChR is an ubiquitous receptor in the CNS, and, therefore, any moiety targeting it holds potential for whole-brain delivery [172]. That is why one such receptor would be potentially adequate for “anti-LSDs” drug delivery, as it may target the neurological phenotype of these diseases. Moreover, RDP may be chemically modified to present more favorable pharmacokinetics. For example, the rabies virus peptide derivative RVG-9r, one of the most widely used RDPs, results from the combination of a short peptide, derived from the RVG that specifically binds nAChRs of neuronal cells, with a sequence of 9 arginines added to its carboxy terminus, which enhances cell uptake and cargo release into the cytoplasm. When complexed with siRNAs, RVG-9r allowed specific delivery of siRNAs to GFP-expressing neuronal cells in vitro, resulting in efficient gene-silencing. In in vivo studies it was demonstrated that following IV injection into GFP transgenic mice, RVG-9r-bound siRNAs were successfully targeted to neuronal cells and promoted efficient and brain-specific gene-silencing [176]. Kumar et al. have also shown that intravascular delivery of RVG-9r-bound antiviral siRNAs in mice was able to confer efficient protection against fatal viral encephalitis. Not accidentally, the same pathway that allows for the transport of RVG throughout the CNS (axonal retrograde transport; [177]) is also responsible for the passage of other neurotoxins, such as the tetanus toxin [178] justifying why the Tet-1 peptide from the tetanus toxin has been looked as a targeting peptide for the delivery of therapeutic nucleic acids to neural cells, in an in vivo study in mice [179]. Likewise, the capacity of the Human Immunodeficiency Virus (HIV) gp120 protein to bind nAChR and the ability of α-bungarotoxin and HIV Tat proteins to cross the BBB, further suggest that peptides derived from other viruses that infect the brain (including HIV, the herpes simplex virus, and flaviviruses) may also be explored as targeting agents for siRNA delivery [175]. To the best of our knowledge, however, so far, these possibilities have not yet been tested. Contrarily, evidence collected on the use of RVG and its derivatives is far more compelling, as reflected in the much higher number of studies using those moieties [173,176]. Therefore, we consider that using one of the RVG peptide derivatives, namely RVG-9r, is particularly tempting for brain delivery of siRNA drugs towards gSRT in LSDs.

It cannot be ignored that there are many other possible ways to promote siRNA brain delivery and several moieties that could be tested for the same purpose as for example lactoferrin [180], folic acid [181,182], glutamate [183], mannose [184] and recombinant IL-1 receptor antagonist [185], among others (reviewed in [186]).

In this regard, our team has shown that covalent coupling of RVG-9r to SNALPs encapsulating siRNAs targeting mutant ataxin-3, which was stably expressed in cultured neuronal cells, promoted specific gene-silencing [187]. Most importantly, IV injection of the RVG-9r-targeted SNALPs-formulated siRNAs into Machado-Joseph transgenic mice expressing mutant ataxin-3 led to efficient silencing of the pathogenic protein, which resulted in drastic reduction of the neuropathology and associated motor behavior abnormalities [187]. Nevertheless, an improved cellular internalization is not necessarily synonymous of an efficient gene-silencing (reviewed in [103,188]), which implies that targeted SNALP-formulated siRNAs must be tested for their efficacy in vivo, even when the effect of the naked molecule has already been extensively analyzed in vitro. Ideally, the effect of these targeted SNALP-formulated therapeutic molecules should be addressed at different levels: mRNA; protein and substrate accumulation and/or excretion. As other authors have already pointed out, in vitro and in vivo models are complementary, and their use must be done heeding limitations of each model. Cell culture models are useful for proof-of-concept studies if an adequate cell type is selected. Currently, the possibility of deriving diverse cell types from affected patients’ iPSCs offers an interesting alternative for siRNA drug testing in different cellular environments. Although in general delivery studies in cell culture have provided very useful data on cellular uptake mechanisms, the results cannot be directly extrapolated to in vivo studies, which are indeed irreplaceable [160].

It is also important to stress, that although we focused this review on SNALPs alone, several other platforms may be used to translate the therapeutic potential of RNAi and deliver its effectors to different organs and tissues. Recombinant adeno-associated vectors (AAV), for example, are among those platforms. In fact, various noncoding RNAi effectors such as shRNAs, miRNAs and siRNA decoys can be expressed from recombinant AAV and there is a significant number of pre-clinical studies evaluating this possibility, which have been extensively reviewed elsewhere [189]. Still, we can anticipate that viral delivery renders the toxicology studies of a product more complex than a classic formulation, which may hinder translational studies with AAV-mediated RNAi vectors. Also, in the nanotechnology field attempts to develop effective delivery systems that protect the siRNA from nuclease degradation, deliver it to target cells, and release it into their cytoplasm, without creating adverse effects are countless. There is an ever-growing number of hypotheses being tested that go far beyond those reviewed here, including dendrimers, inorganic nanoparticles, exosomes, extracellular vesicles, and even red blood cells. Interestingly, some of these approaches are actually being attempted in the LSDs field, but not for siRNA delivery. For example, both AAV vectors [190] and extracellular vesicles [191] have been tested for their potential to treat CLN2 Batten disease acting as drug carriers for gene therapy or ERT, respectively.

Finally, there is one last issue that deserves attention to improve siRNA delivery into its target tissues: administration. Currently, there are several possible approaches, from IV injection to more direct delivery methods, including ICV or IT injection. The systemic route for delivery into the CNS includes diffusion [192] and RME [193,194] and requires the development of a siRNA nanodelivery system, which is able to cross the BBB, such as the RVG-targeted SNALPs we have been referring to. So far, however, the most widely used methods to bypass the BBB are the direct brain parenchyma injections, which allow for the use of non-targeted nanocarriers or chemically modified naked therapeutic molecules.

Commonly, delivery optimization is often left until the latter stages of pre-clinical development, a practice that can considerably delay the successful clinical translation of molecular therapies (reviewed in [160]). The LSDs field is no exception t, with a scarce number of in vitro proof-of-principle studies on different molecular approaches to either overcome or minimize pathology [66,94,95,96,97,98,195]; barely contributed to enrich the knowledge of the development of proper therapeutic delivery methods. As far as we know, there are no studies on the development of targeted SNALPs for brain delivery of siRNAs designed to decrease substrate biosynthesis in LSDs, but the potential of one such approach does seem undeniable. Among other important advantages, one such approach would virtually allow for the generation of multifunctional siRNA mixtures that could be applied to different diseases, as they share the same accumulating substrates, thus reducing therapy costs and increasing the number of patients that could benefit from available therapeutic options.

## 7. Concluding Remarks and Future Perspectives

Since the discovery of the Nobel prize-winning mechanism of RNAi, 20 years ago, this technology has become not only a major gene-silencing tool in the lab for the experimental manipulation of gene expression, but also a promising therapeutic approach. Since it is a naturally occurring post-transcriptional process, this silencing mechanism has several advantages when compared to other technologies. That recognition triggered major investments in RNAi-based drug development by large pharmaceutical and biotechnological companies, which culminated in the first U.S. FDA approval of one such drug in 2018 (reviewed in [6]).

In general, the clinical application of this sort of drugs largely depends on the development of appropriate delivery systems, which are able to ameliorate the unfavorable pharmacokinetics of RNAi effectors (siRNAs), while enhancing their biodistribution properties. Throughout these decades, many different types of nanomaterials have been developed for siRNA delivery, and based on the extensive evaluation of their properties, lipid-based nanoparticles have probably become the most popular ones. Among them, PEG-grafted nanocarriers and, particularly, SNALPs, have gained momentum (reviewed in [80,103]) in such a way that SNALPs represent one of the most widely adopted RNAi delivery technology to date. At present, human applications of SNALP-formulated siRNAs are yet almost exclusively confined to targets within the liver, where siRNA nanodelivery systems naturally accumulate. Extra-hepatic targets remain a challenge [196], but expectantly as we have here reviewed, there are several in vivo studies addressing these targets, whose positive results hold promise for a wider application of SNALPs in other disorders, including those that affect the CNS. LSDs are certainly among those disorders, as they may benefit from brain-targeted gSRT, an approach that can circumvent one of the most urgent unmet medical needs for these patients: CNS pathology and the relentless neurodegeneration, which ultimately results in premature death.

## Abbreviations

AGO2	Argonaute 2
ApoB	Apolipoprotein B
ASOs	Antisense oligonucleotides
BBB	Blood-brain barrier
C6-Cer	C6-ceramide
CML	Chronic myeloid leukemia
CNS	Central nervous system
CSCs	Cancer stem cells
CSF	Cerebrospinal fluid
CTX	Chlorotoxin
DAGs	Diacylglycerols
DSPC	1,2-distearoyl-sn-glycero-3-phosphocholine
dsRNA	Double-stranded RNA
DXR	Doxorubicin
EBOV	Ebola virus
ERT	Enzyme replacement therapy
FD	Fabry disease
GAGs	Glycosaminoglycans
GD	Gaucher disease
GBM	Glioblastoma
gSRT	Genetic substrate reduction therapy
hATTR	Hereditary TTR-mediated amyloidosis
HBV	Hepatitis B virus
HF	Hemorrhagic fever
HIV	Human immunodeficiency virus
HMEC-1	Angiogenic endothelial cells
HS	Heparan sulphate
ICV	Intracerebroventricular
iPSCs	Induced pluripotent stem cells
IT	Intrathecal
IV	Intravenous
KSP	Kinesin spindle protein
LNPs	Lipid nanoparticles
LDL	Low-density lipoprotein
LSDs	Lysosomal storage disorders
miRNA	microRNA
MLD	Metachromatic Leukodystrophy
MPSs	Mucopolysaccharidoses
mRNA	messenger RNA
NCAM	Neural cell adhesion molecule
NCLs	Neuronal Ceroid Lipofuscinoses
nACh	Nicotinic acetylcholine
nAChR	Nicotinic acetylcholine receptor
n-SCC	Non-stem cancer cells
PD	Parkinson’s disease
PLK1	Polo-like kinase 1
PEG	Polyethylene glycol
PEI	Polyethylenimine
PC3	Prostate cancer cells
PKR	Protein kinase receptor
P75NTR	Neurotrophin receptor 75
RDP	Rabies virus derived peptide
RVG	Rabies virus glycoprotein
RVG-9r	Rabies virus peptide derivative
RME	Receptor-mediated endocytosis
rhTPP1	Recombinant human tripeptidyl peptidase 1
RES	Reticuloendothelial system
RNAi	RNA interference
RISC	RNA-induced silencing complex
shRNA	Short hairpin RNA
siRNAs	small interfering RNAs
SNALP	Stable nucleic acid lipid particle
SRT	Substrate reduction therapy
TLRs	Toll-like receptors
Trf	Transferrin
TrfR	Transferrin receptor
TTR	Transthyretin
T_1/2_	Elimination/circulation half-time
U.S. FDA	United States Food and drug administration
VLDL	Very-low-density lipoprotein
ZEBOV	Zaire species of EBOV
3’-UTR	3’ untranslated region
